# Efficacy of chitosan paste as intracanal medication against *Enterococcus faecalis* and *Candida albicans* biofilm compared with calcium hydroxide in an in vitro root canal infection model

**DOI:** 10.1186/s12903-022-02385-x

**Published:** 2022-08-16

**Authors:** Pasika Thienngern, Anchana Panichuttra, Chootima Ratisoontorn, Chuanchom Aumnate, Oranart Matangkasombut

**Affiliations:** 1 Department of Operative Dentistry, Faculty of Dentistry, Chulalongkorn University, 34 Henri- Dunant Road, Wangmai, Patumwan, Bangkok, 10330 Thailand; 2 Metallurgy and Materials Science Research Institute, Chulalongkorn University, Soi Chula 12, Phayathai Road, Pathumwan, Bangkok, 10330 Thailand; 3 Department of Microbiology and Center of Excellence on Oral Microbiology and Immunology, Faculty of Dentistry, Chulalongkorn University, 34 Henri- Dunant Road, Wangmai, Patumwan, Bangkok, 10330 Thailand; 4 Research Laboratory of Biotechnoloty, Chulabhorn Research Institute, Kampangpetch 6 Rd, Laksi, Bangkok, 10120 Thailand

**Keywords:** *Enterococcus faecalis*, *Candida albicans*, Chitosan, Intracanal medication, Polyethylene glycol, Propylene glycol

## Abstract

**Background:**

*Enterococcus faecalis* and *Candida albicans* are frequently found in persistent endodontic infection and could remain in dentinal tubules despite intracanal medication with calcium hydroxide (Ca(OH)_2_), a commonly used medication. Thus, an effective and safe antimicrobial medication against such refractory infection is necessary in endodontic retreatment, so we aimed to test the efficacy of chitosan paste against these microorganisms compared with Ca(OH)_2_ in root canals of extracted human teeth.

**Methods:**

Thirty-six sterilized human root samples prepared from extracted premolars and upper maxillary incisors were infected with *E. faecalis* for 14 days, while 32 were infected with *C. albicans* for 48 h, for mature biofilm formation. The samples were assigned to 6 groups of intracanal medications: Group 1: no medication (negative control); Group 2: 20% Polyethylene glycol (PEG); Group 3: 20% Propylene glycol (PG); Group 4: Ca(OH)_2_; Group 5: Chitosan + PEG; and Group 6: Chitosan + PG. After 7 days, intracanal surface dentin was harvested using Protaper next, resuspended, serially diluted and spread on Brain–Heart-Infusion agar (for *E. faecalis)* and Yeast Extract-Peptone-Dextrose agar (for *C. albicans*) for colony count. Antimicrobial efficacy was determined as percentage of remaining colony forming unit (CFUs) relative to negative control and analyzed using One-way ANOVA and post-hoc Games-Howell test. The significance level was set at 0.05.

**Results:**

For *E*. *faecalis,* chitosan + PG had significantly higher antibacterial activity than Ca(OH)_2_ (*P* = 0.039). Chitosan + PEG and chitosan + PG medication significantly reduced viable bacteria compared with negative control, PEG and PG (*P* = 0.001, 0.003, 0.024, respectively for chitosan + PEG; *P* = 0.002, 0.003, 0.014, respectively for chitosan + PG). For *C.albicans,* chitosan + PEG and chitosan + PG were not significantly different from Ca(OH)_2_. However, Chitosan + PEG and chitosan + PG, but not Ca(OH)_2_, showed a significantly lower level of remaining CFUs compared with negative control (*P* = 0.013 and 0.005, respectively).

**Conclusion:**

Chitosan paste showed better efficacy in reducing viable *E. faecalis* biofilm when compared to Ca(OH)_2_ after 7-day intracanal medication in this in vitro root canal model. It could also significantly reduce viable *C. albicans,* but was not significantly different from Ca(OH)_2_.

**Supplementary Information:**

The online version contains supplementary material available at 10.1186/s12903-022-02385-x.

## Background

A major factor in endodontic failure in root canal-treated teeth is persistent infection [1, 2]. Thus, it is critical to have effective disinfection of the root canal system. To eradicate microorganisms, intracanal medication is an important part of endodontic treatment that relies on the efficacy of antimicrobial agents [2]. Calcium hydroxide (Ca(OH)_2_) has been used as a routine intracanal medication. However, certain microorganisms are still frequently detected in failed endodontic treated teeth, such as *Enterococcus faecalis* and *Candida albicans* [3]*.*

*E. faecalis* is a Gram positive cocci that can invade dentinal tubules, form biofilm, attach to collagen in serum, and suppress lymphocyte activity, which can protect them from destruction [4]. It also has a proton pump inhibitor mechanism to resist to a wide pH range, up to approximately pH 11.5 [5]. Thus, it can tolerate the alkalinity of Ca(OH)_2_, could remain after root canal obturation, and is frequently found in persistent infection [3, 6]. *C. albicans* is another microorganism that has been reported in persistent post-treatment apical periodontitis [7]. An important virulence factor of *C. albicans* is the ability to switch between blastospore and hyphal form. This enables it to invade host tissue and avoid phagocytosis by macrophages [8]. Thigmotropism allows *C. albicans* to penetrate into deep dentinal tubules [9]. *C. albicans* can form biofilm in 48 h [10]. It can survive in a wide range of pH, high alkaline environment and ecologically harsh conditions, which allow them to cause persistent infection [8].

Chitosan is a natural polysaccharide derived from deacetylation of chitin in crustacean shells. Chitosan has antimicrobial, antifungal properties and enhances wound healing [11]. Chitosan can interact with microbial outer cellular components, cell membrane, and cytoplasmic constituents, and could inhibit biofilm formation [12]. Chitosan also has high biocompatibility, low toxicity, and showed inhibitory effect on planktonic form and biofilm of *E. faecalis* and *C. albicans* [13, 14]; thus, it may be effective as an antimicrobial intracanal medication. Previously published studies have shown that certain derivatives of chitosan are effective against *E. faecalis* and common oral *Candida* species, including *C. albicans* [15–17]. In particular, it has been demonstrated that 1700 kDa and 2100 kDa chitosan are effective against *E. faecalis,* but they require a long contact time (over 1 h) [17]. Thus, we propose that these chitosan derivatives are promising to be developed into intracanal medication [17, 18].

In order to formulate chitosan into intracanal medication, Polyethylene glycol (PEG) and Propylene glycol (PG) can be used as vehicles to deliver intracanal medication through dentinal tubules and apical foramen [19]. In addition, a previous study suggested that propylene glycol have antibacterial effect on *S. mutans, E. faecalis*, and *E. coli* [20]. Thus, it may also contribute to antimicrobial activity of the medication. This study aimed to develop chitosan paste as intracanal medication and test its antimicrobial activity against *E. faecalis* and *C. albicans* in comparison with Ca(OH)_2_ in the root canals of extracted human teeth. The null hypothesis was that chitosan paste would not be different from Ca(OH)_2_ in terms of antimicrobial activity against *E. faecalis* and *C. albicans.*

## Methods

This study was carried out in accordance with Declaration of Helsinki and the proposal was approved by "The Human Research Ethics Committee of the Faculty of Dentistry, Chulalongkorn University, Bangkok, Thailand" (No. 055/2020). Informed consent was waived because the donors of the extracted tooth samples were unidentifiable.

### Root samples

Sixty-eight intact premolars and upper maxillary incisors with single straight root canal extracted for orthodontic and periodontitis reasons were collected. Teeth with caries, **f**ractures, cracks or other defects detected by magnifying loupes were excluded. The tooth samples were prepared as described with minor modification [21, 22]. All extracted teeth were stored in 0.1% thymol (MU DENT, Mahidol university, Bangkok, Thailand) until prepared for the experiments [22]. All soft-tissue remnants on the surfaces, the crowns and the coronal third of roots were removed until each root was 15 mm long. The root canals were enlarged by using Protaper next size X4 (Dentsply Sirona, Ballaigues, Switzerland) with 300 RPM speed and 2 g cm torque in a rotary handpiece. The samples were irrigated with 3 ml of 2.5% sodium hypochlorite (NaOCl) (Faculty of Dentistry, Chulalongkorn University, Bangkok, Thailand) followed by 1 ml of 17% ethylenediaminetetraacetic acid (EDTA) (Faculty of Dentistry, Chulalongkorn University, Bangkok, Thailand) to remove organic and inorganic debris. All samples were irrigated with 5 ml of distilled water to remove any remaining prior irrigants, and autoclaved for 20 min at 121 °C. The external surfaces were sealed with nail vanish. The samples were divided into 2 groups, 36 were inoculated with *E. faecalis* and 32 were inoculated with *C. albicans.* We calculated sample size based on a previous report on the differences in viable bacterial count between calcium hydroxide and calcium hydroxide combined with chitosan nanoparticles [21] using n4studies application [23]. The calculated sample size was 2 samples per group at 80% power of test. Nevertheless, all experiments were performed three times, each with 1–2 samples/group, so the total sample size was 5–6/group.

### Preparation of microbial culture

*E. faecalis* (ATCC 29212) was incubated in brain heart infusion (BHI) broth (Himedia, Mumbai, India) at 37 °C until log phase. *C. albicans* (ATCC 90028) was incubated in Yeast extract—peptone-dextrose broth (YPD; Oxoid, UK and HiMedia, India) at 30 °C until log phase. The microbial suspension was adjusted to optical density of 0.5 for *E. faecalis* and 0.1 for *C. albicans* at 600 nm for inoculation into the root canals.

### Infection of the root samples

Log phase culture (30 μl) of *E. faecalis* or *C. albicans* was inoculated into the root canals. For *E. faecalis,* BHI media was replenished every 48 h and the samples were incubated for 14 days for mature biofilm formation [24]. For *C. albicans*, the root samples were incubated for 48 h for mature biofilm formation [10]. All procedures were carried out in a biosafety cabinet (LabGard NuAire Inc, MN, USA).

### Preparation of intracanal medications

The chitosan powder (1700 KDa, Marine Bio Resources, Samutsakhon, Thailand) was dissolved in 1% acetic acid (Merck KGaA, Darmstadt, Germany) at 20 mg/ml. The chitosan pastes were prepared by mixing 1 ml of chitosan solution (20 mg/ml) with 1 ml of Polyethylene glycol (PEG; Krungthepchemi, Bangkok, Thailand) or propylene glycol (PG; Krungthepchemi, Bangkok, Thailand), and 3 ml of distilled water. The final concentration of chitosan was 4 mg/ml in 20% PEG or PG. The pastes were sterilized by autoclave (Hirayama, Tokyo, Japan).

Ca(OH)_2_ intracanal medication was prepared by mixing 0.4 g of Ca(OH)_2_ powder (Faculty of Dentistry, Chulalongkorn University, Bangkok, Thailand) with 10 ml of distill water. The final concentration of Ca(OH)_2_ was 40 mg/ml.

### Antimicrobial assessment

After the specified incubation period, an aliquot of the media from each sample was plated on solid media to check for microbial purity and viability. Root samples infected with *E. faecalis* and those with *C. albicans* were allocated to 6 groups and treated as follow Group 1: no medication (negative control), Group 2: 20% Polyethylene glycol (PEG), Group 3: 20% Propylene glycol (PG), Group 4: Ca(OH)_2_, Group 5: Chitosan + PEG, and Group 6: Chitosan + PG. Thirty μl of the assigned medication were applied into each root canal using a micropipette (Rainin Instrument Co., Oakland, CA, USA).The whole volume was delivered to ensure that the canal was filled. The root samples were incubated at 37ºC for *E. faecalis* and 30ºC for *C. albicans* in 24-well plates with covers for 7 days. After 7 days, the canals were washed with 3 ml of sterile distilled water and Protaper next size X4 was used at 300 RPM speed and 2 g cm torque to remove the medicament.

Dentin samples were harvested from root canal surfaces as previously described with minor modification [25] using Protaper next size X5 (Dentsply Sirona, Ballaigues, Switzerland) at 300 RPM speed and 2gcm torque and collected in 1 ml of phosphate buffered saline solution. After serial dilutions, 100 μl of each dilution were plated on BHI agar for *E. faecalis* and YPD agar for *C. albicans*, and incubated for 24 h at 37 °C for *E. faecalis* and 48 h at 30 °C for *C. albicans*. Colonies were counted and calculated into percentage of remaining viable microorganisms relative to the negative control. All experiments were performed in duplicates and repeated 3 times (except for the PEG and PG groups for *C. albicans* where only one sample/group was available for two of the experiments).

### Statistical analysis

Shapiro–Wilk was used to test for normality of the data. Welch’s ANOVA was used to analyze the differences in percentage of remaining viable microorganisms after treatment among groups, followed by Games-Howell test for pairwise comparison. Data were analyzed using IBM SPSS Statistics for Windows, Version 22.0 (IBM, Armonk, New York, USA). A *P* value of < 0.05 was considered statistically significant.

## Results

**Fig. 1:**
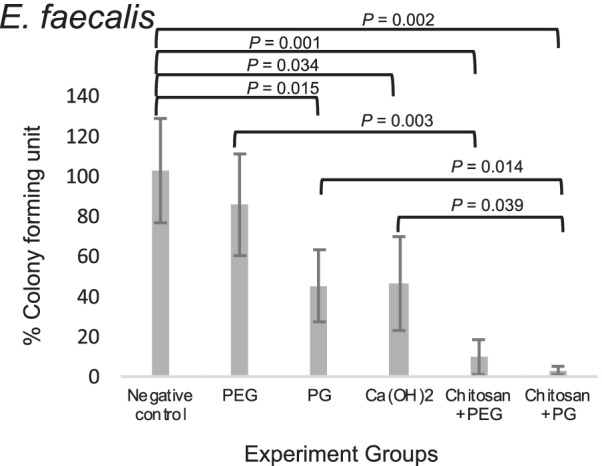
Percentage of remaining viable *E. faecalis* after treatment with intracanal medication relative to negative control

**Fig. 2:**
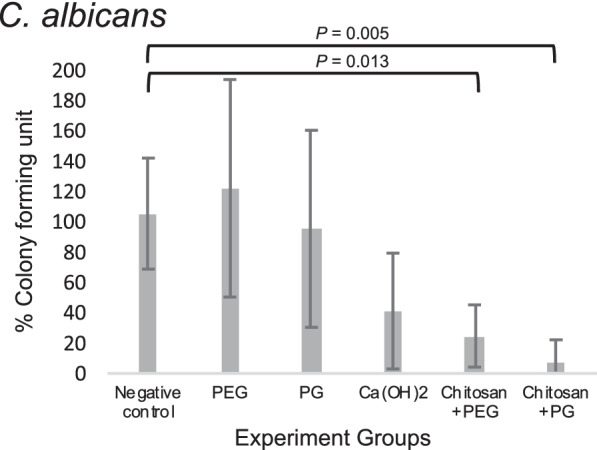
Percentage of remaining viable *C. albicans* after treatment with intracanal medication relative to negative control

After 7 days of treatment with various medications, the remaining viable microorganisms in the root canals were examined by plate count. The results are shown in Fig. 1 for *E. faecalis* and Fig. 2 for *C. albicans*. For *E. faecalis,* the average percentage of remaining bacteria in both chitosan + PEG (9.68 ± 8.6%) and chitosan + PG (3.02 ± 2%) groups was significantly lower than that of the negative control (102.74 ± 26%), PEG (85.7 ± 25.3%) and PG (45.28 ± 17.9%) groups. (*P* = 0.001, 0.003, and 0.024, respectively for chitosan + PEG; *P* = 0.002, 0.003, and 0.014, respectively for chitosan + PG). In addition, the PG group showed a significantly lower remaining *E. faecalis* than the negative control (*P* = 0.015). Ca(OH)_2_ (46.38 ± 23.4%) also had a significantly lower level of remaining *E. faecalis* than the negative control group (*P* = 0.034). Chitosan + PG could reduce the bacteria to a level that is significantly lower than the Ca(OH)_2_ group (*P* = 0.039) but this was not significantly different for the chitosan + PEG group (*P* = 0.071).

For *C. albicans,* both chitosan + PEG (24.77 ± 20.5%) and chitosan + PG (7.57 ± 14.6%) groups harbored significantly lower remaining viable *C.albicans* compared with the negative control (105.40 ± 36.5%) (*P* = 0.013 and 0.005, respectively). However, they were not significantly different from PEG (122.06 ± 71.7%) and PG (95.36 ± 64.8%) groups (*P* = 0.292, and 0.441, respectively for chitosan + PEG; *P* = 0.206 and 0.293, respectively for chitosan + PG). In contrast, PEG, PG, and Ca(OH)_2_ (41.19 ± 38.1%) were not significantly different from the negative control (*P* = 0.997, 1.00, and 0.106, respectively).

## Discussion

In this study, we showed that 1700 kDa chitosan (4 mg/ml) paste has good antimicrobial efficacy against *E. faecalis* and *C. albicans* biofilm in human root canals, especially chitosan + PG paste, which showed significantly higher efficacy than calcium hydroxide against *E. faecalis.* The results suggest that chitosan paste is a promising candidate to be further developed into an antimicrobial intracanal medication, particularly in cases with persistent *E. faecalis* infection.

Microbiological investigations found a complex community of bacteria and fungi in root canal treated teeth with persistent infection/chronic apical periodontitis [1, 3]. This suggests that previous endodontic treatment could not efficiently control these microorganisms. Mechanical instrumentation using larger size rotary instruments may help to remove bacteria and fungi in the root canals, but it cannot completely eradicate microbes in complex root canal structures and excessive instrumentation weakens root dentin [26–29]. Thus, effective antimicrobial intracanal medication can overcome the limitations of instrumentation to reduce microorganisms in complex anatomy of the root canals. Ca(OH)_2_ has been used as routine intracanal medication, but it was not effective against *E. faecalis* and *C. albicans* [30–32]*.* Several studies have identified *E. faecalis* and *C. albicans* as important microorganisms using culture-dependent and molecular techniques [3, 33, 34]. For example, a recent study used culture-dependent methods and showed that the most prevalent microorganisms in root canal treated teeth was *E. faecalis* (36.6%) and followed by *C. albicans* (20%) [3]. Studies using PCR detected *E. faecalis* at a prevalence of up to 77%, and *C. albicans* up to 35%, of failed root-filled teeth [33, 34]. Although *E. faecalis* was not the most abundant bacteria detected by metagenomic studies, it was observed at a greater frequency or proportion in secondary apical periodontitis than primary infection [35, 36]. Thus, persistence of these microorganisms in the root canals are problematic for endodontic treatment and effective antimicrobial agent against *E. faecalis* and *C. albicans* in root canals is clearly needed [32, 37, 38].

Our group has previously shown that 1700 kDa chitosan and 2100 kDa chitosan could effectively kill *E. faecalis* at an MBC of 2 mg/ml [17]. However, it needed a long contact time of over 10 min, so it is likely more effective when applied as a root-canal medication rather than as an irrigant. In addition, we also showed that these chitosan derivatives has a minimum fungicidal concentration against *C. albicans* at 4 mg/ml [16]. Thus, in this study, we formulated 4 mg/ml of 1700 KDa chitosan in 0.2% acetic acid into a paste for application as an antimicrobial intracanal medication. We used 20% PEG or PG to confer good flowability for easy handling and enhance penetration into dentinal tubules.

Our results showed that chitosan paste could eliminate more *E. faecalis* than negative control and carrier controls. Chitosan + PG showed greater antibacterial effect against *E. faecalis* biofilm than PG, and also greater than Ca(OH)_2_. Although *E. faecalis* could resist to Ca(OH)_2_ by a proton pump mechanism, our result showed that Ca(OH)_2_ was better at reducing viable *E. faecalis* than no medication [30]. Since PG could also reduce *E. faecalis* compared with negative control, the combined effect of both chitosan and PG may explain why chitosan + PG is the most effective in this experiment. Chitosan is positively charged and binds the negatively charged microbial cell membrane, while PG can help intracanal medication to penetrate deeper in dentinal tubules and it also has germicidal activity [20, 39, 40]. Similarly, chitosan-propolis nanoparticles was shown to be effective at eliminating *E. faecalis* biofilm after 7 days of medication [18]. Thus, chitosan is a promising alternative option for intracanal medication, either alone or in combination with other active ingredients, especially in infection where *E. faecalis* and/or *C. albicans* infection may play important roles. We also found that chitosan paste was effective against *C. albicans* biofilm as both chitosan groups had significantly less remaining viable *C. albicans* than the negative control. We could not detect significant differences among other groups, likely due to the wide variations in the CFU results. For *C.albicans*, Ca(OH)_2_ was not significantly different from the negative control group. Our results suggest that Ca(OH)_2_ is more effective against *E. faecalis* than *C. albicans.* This is concordant with a report by Ercan and colleagues [41].

Microorganisms in biofilm are more tolerant to antimicrobial agents, and this is the form found in the root canals [8, 42]. Thus, we simulated such biofilm condition in this study using extracted human root specimens inoculated with *E. faecalis* and with *C. albicans,* and allowed sufficient time for mature biofilm formation and penetration in dentinal tubules, i.e., 14 days for *E. faecalis* and 48 h for *C. albicans,* according to previous reports [10, 42, 43]. In addition, we collected dentin shavings to evaluate remaining viable microorganisms. In contrast to sample collections by irrigation or using paper points, this method allowed us to examine bacteria that invaded into dentinal tubules that may not have direct contact with intracanal medication if it does not penetrate well into dentinal tubules [44]. Our results showed that chitosan paste could significantly reduce viability of both *E. faecalis* and *C. albicans* in this root canal biofilm model. Thus, it has a great advantage over antibiotics medication, which are effective only against bacteria, but not fungi, and may also lead to tooth discoloration [45]. Nevertheless, this in vitro model used single-species biofilm, unlike in vivo conditions where multiple species coexist in a community. Therefore, further studies on multi-species biofilm and clinical trials should be performed in order to develop chitosan paste for future clinical applications. In addition, in this study, the effects of the medication were examined at 7-day after medication. This may be a limitation because previous studies suggested that a longer period of medication may be required [46]. However, a previous study showed that the antimicrobial activity of Ca(OH)_2_ after 7 day and 14-day medication was not significantly different [47] and another study showed that pH of Ca(OH)_2_ already increased after 7 days [48]. Nevertheless, further research on optimizing the length of medication time will be required.

## Conclusion

In this root-canal biofilm model, chitosan paste showed better efficacy in reducing viable *E. faecalis* biofilm when compared to Ca(OH)_2_ after 7-day intracanal medication in this in vitro root canal model. It also significantly reduced viable *C. albicans,* but was not significantly different from Ca(OH)_2_. Therefore, it could be developed into an effective antimicrobial intracanal medication".

## Supplementary Information

**Additional file 1.** Percentage of remaining viable CFU of *E. faecalis* and *C. albicans* relative to control in 3 independent experiments.

## Data Availability

The data analyzed in the present study are available in Additional file 1.

## Abbreviations

Ca(OH)_2_: Calcium hydroxide
*E. faecalis*: *Enterococcus faecalis*
*C. albicans*: *Candida albicans*
PEG: Polyethylene glycol
PG: Propylene glycol
ANOVA: Analysis of variance
